# Evaluation of the effectiveness regarding the participation of pharmacists in perioperative blood glucose management via the iGMS: a pilot RCT

**DOI:** 10.1186/s13098-023-01221-8

**Published:** 2023-11-17

**Authors:** Jinfang Song, Xiaojun Pan, Ya Chen, Yongjuan Ding, Xia Li

**Affiliations:** 1 Department of Pharmacy, Affiliated Hospital of Jiangnan University, No.1000, Hefeng Road, Wuxi, Jiangsu 214000 China; 2 Jiangsu Key Laboratory of New Drug Research and Clinical Pharmacy, Xuzhou Medical University, Xuzhou, 221000 China; 3 Department of Pharmacy, Wuxi No. 5 People’s Hospital, Wuxi, 214000 China; 4 Department of Endocrinology, Affiliated Hospital of Jiangnan University, Wuxi, 214000 China

**Keywords:** Clinical pharmacist, iGMS, Perioperative period, Type 2 Diabetes Mellitus

## Abstract

**Background:**

Excellent blood glucose management is a key guarantee for successful progress of surgery. However, the impact of clinical pharmacists on blood glucose management of perioperative patients needs to be further investigated. To investigate the effectiveness regarding the participation of pharmacists in blood glucose management via the informatized glucose management system (iGMS) on perioperative patients with type 2 diabetes mellitus (T2DM).

**Methods:**

The working mode of clinical pharmacists participating in blood glucose management of perioperative patients with diabetes was constructed. A total of 300 patients with T2DM who underwent elective surgery were recruited and divided into a clinical pharmacist management group (intervention group) of 150 patients (94 men and 56 women; mean age: 44.38 ± 14.03 years) and a control group of 150 patients (101 men and 49 women; mean age: 47.85 ± 12.26 years) between September 2019 to April 2020. The outcomes of perioperative blood glucose management, and healthcare indicators such as preoperative waiting time, total hospitalization time, postoperative infection rate and other indicators were analyzed statistically between the two groups.

**Result:**

In the blood glucose management team of the whole hospital, the physicians, clinical pharmacists and nurses of blood glucose management in endocrinology department were the core members, and were responsible for perioperative blood glucose management of the participants in the intervention group. All subjects had lower blood glucose after 3 days of management compared to the time of admission, and blood glucose was significantly lower in the intervention group compared to the control group (*P* < 0.05). As compared with the control group, subjects in intervention group demonstrated significant differences in outcome measures. The relevant parameters included preoperative blood glucose compliance rate (60.67% vs. 35.33%, *P*<0.05), preoperative waiting time [(5.27 ± 3.34) vs. (7.45 ± 4.38), *P*<0.05], length of hospitalization [(11.11 ± 4.56) vs. (14.87 ± 5.39), *P*<0.05], incidence of hypoglycemia (8.67% vs. 18.00%, *P*<0.05), incidence of hyperglycemia (32.00% vs. 62.67%, *P*<0.05) and postoperative infection rate (18.00% vs. 24.67%, *P* > 0.05).

**Conclusion:**

The involvement of clinical pharmacists in blood glucose management utilizing the iGMS can control the blood glucose level of patients with T2DM in the perioperative period more stably and effectively, thereby leading to an improvement in the quality of healthcare.

**Supplementary Information:**

The online version contains supplementary material available at 10.1186/s13098-023-01221-8.

## Background

According to the data of the Diabetes Atlas 2021 (10th edition) released by the International Diabetes Federation, the number of adult diabetes patients in the world will reach 537 million in 2021 (accounting for 10.5% of the total population, up 16% from 2019), and it is estimated that by 2045 this number will reach 783 million [1]. The prevention and treatment of diabetes and its complications **have** become a major health problem for the whole society. More importantly, studies have shown that approximately 50% of patients with diabetes undergo at least one surgical procedure in their lifetime, while up to 75% of patients with diabetes over 50 years old have had a surgical experience, and nearly 20% of patients receiving surgery have diabetes [2, 3]. Diabetes and surgery are prone to have significant interaction. Perioperative stress can exacerbate the hyperglycemic state, leading to abnormalities in body fat, protein, and carbohydrate metabolism as well as disorders of the immune system, increasing the risk of adverse outcomes such as cardiovascular complications, anesthesia accidents, and impaired tissue regeneration and repair [4–6]. On the other hand, abnormally elevated blood glucose in the diabetic state is also capable of causing a variety of adverse clinical outcomes in surgical patients, such as delayed wound healing time, increased incidence of postoperative infections, and prolonged hospitalization [7–9]. Stephen et al. [10] found that the rate of hospital-acquired infections in patients with postoperative blood glucose > 12 mmol/L increased 5.7-fold for elective surgery. Yeh et al. [11] showed that patients with diabetes mellitus have a higher risk of acute kidney dysfunction postoperatively. Hypoglycemia is also a crucial manifestation of perioperative blood glucose abnormalities, and the consequences of hypoglycemia are typically more severe than those of hyperglycemia [12, 13]. Due to the residual effects of anesthesia or sedative drugs in the body after surgery, hypoglycemic episodes often come with symptoms like altered consciousness and drowsiness. This can make it challenging to detect hypoglycemic symptoms, potentially leading to misleading healthcare professionals and missing the opportunity for timely intervention, thereby increasing the mortality rate of critically ill patients. Therefore, excellent blood glucose management is a key guarantee for successful progress of surgery and the safety of patients in the perioperative period, and standardized management of blood glucose in the perioperative period is crucial for the prognosis of patients with diabetes mellitus [14]. For surgical patients with abnormally high blood glucose during hospitalization or diagnosed diabetes, the traditional mode of blood glucose management for patients is to invite endocrinologists to consult and formulate a hypoglycemic plan, and then surgeons manage the blood glucose of patients. The mindset limitations of non-endocrine specialists have resulted in the lack of attention to glucose monitoring, untimely adjustment of glucose-lowering regimens, and ineffective control of the risk of hypoglycemia in perioperative diabetic patients. A new model of glycemic management is highly desirable in order to provide timely, rapid, accurate, and standardized treatment regarding blood glucose in patients with diabetes mellitus during the perioperative period in order to reduce the risk of adverse outcomes.

In recent years, there has been significant development in medical equipment for blood glucose monitoring, leading to the emergence of the informatized glucose management system (iGMS). The iGMS can connect to a network cloud platform, enabling remote real-time data transmission, rapid access to patient records, and blood glucose monitoring critical value alerts, among other functions [15]. The prominent advantages of iGMS are as follows: (1) Healthcare professionals, including doctors, pharmacists, and nurses, can comprehensively and professionally manage blood glucose, ensuring the timeliness and accuracy of blood glucose data. (2) By following the workflow of scanning barcodes, measuring blood glucose, uploading data, and receiving data feedback, efficiency is improved, and errors are reduced. (3) Simultaneously, through the extraction and analysis of blood glucose data, it contributes to the accumulation and summarization of hospital clinical experience, enhancing the quality of healthcare services and facilitating research work [16–18]. Reducing the incidence of severe hyperglycemia and iatrogenic hypoglycemia is a crucial issue for improving the quality and safety of blood glucose management. Studies have shown that the iGMS plays a significant role in promoting blood glucose control [19, 20].

There is evidence that clinical pharmacist can improve the comprehensive management of diabetes mellitus by building doctor-pharmacist joint pharmacy clinic for outpatients [21]. However, the impact of clinical pharmacists on blood glucose management of perioperative patients needs to be further investigated. In this study, a clinical pharmacist-participated perioperative patient blood glucose management system was constructed based on the informatized glucose management system (iGMS), and the perioperative blood glucose management practice of type 2 diabetes mellitus (T2DM) patients was used as an example to explore the management effect. The primary objective of the current study was to evaluate the effectiveness regarding the participation of pharmacists in blood glucose management via the iGMS on perioperative patients with T2DM.

## Methods

### Subjects

From September 2019 to April 2020, this study included patients with T2DM who underwent elective surgery in orthopedic, urologic, general, cardiovascular, cardiothoracic, and gynecologic departments at the Affiliated Hospital of Jiangnan University (Original Third Hospital). According to the method of random number table, a total of 300 participants were divided into control group (101 male and 49 female) and intervention group (94 male and 56 female). Inclusion criteria: (1) meet the diagnostic criteria for T2DM given by the world health organization (WHO) in 1999; (2) meet the indications for surgery; (3) patients and their families give informed consent and can effectively cooperate with the medical professionals. Exclusion criteria: (1) patients with severe respiratory, cardiovascular, or hepatic or kidney diseases; (2) patients with unclear consciousness or communication disabilities; (3) individuals with neurological or psychiatric disorders; (4) patients and their families unable to comprehend the work of blood glucose management team; (5) Minors, pregnant or lactating women; (6) patients with prolonged postoperative fasting and use of nutrient preparations; (7) patients with a preoperative or postoperative management duration shorter than 3 days.

### Model of blood glucose management

The traditional blood glucose management model was performed for the control group of patients. The mobile blood glucose detector was used to measure capillary blood glucose of patients. The patients with T2DM who had been diagnosed, the starting frequency of blood glucose monitoring was 4 times daily. The attending physician invited an endocrinology specialist for a consultation when the FPG ≥ 7.0 mmol/L or PPG ≥ 11.1 mmol/L. The consulting physician formulated a treatment regimen according to the glycemic record sheet and other examination results, and the attending physician followed the medical advice of the consulting physician.

For patients of the intervention group, the clinical pharmacist would manage the blood glucose of the patients via the informatized glucose management system (iGMS, Beijing Huayi Jingdian Biotechnology Co.). A clinical smart glucose meter known as the GLUPAD is applied to monitor the blood glucose levels of patients, and in the same way, the nurse can use the GLUPAD to identify and confirm the patient at the bedside of the patient via a wrist barcode before measuring the blood glucose. After the measurement of blood glucose, the data from the GLUPAD can be automatically synchronized to the iGMS system for storage, archiving and analysis. Clinical pharmacists can access medical history information, medication records, property of disease, dietary status, surgical procedures, bio-chemical outcomes, graphical glycemic fluctuations at various times during the hospitalization, and notes on major glycemic events via the iGMS. Thresholds for hyperglycemia and hypoglycemia are established, and the iGMS is equipped with a reminder function once the blood glucose uploaded is below or above the defined thresholds.

**Fig. 1:**
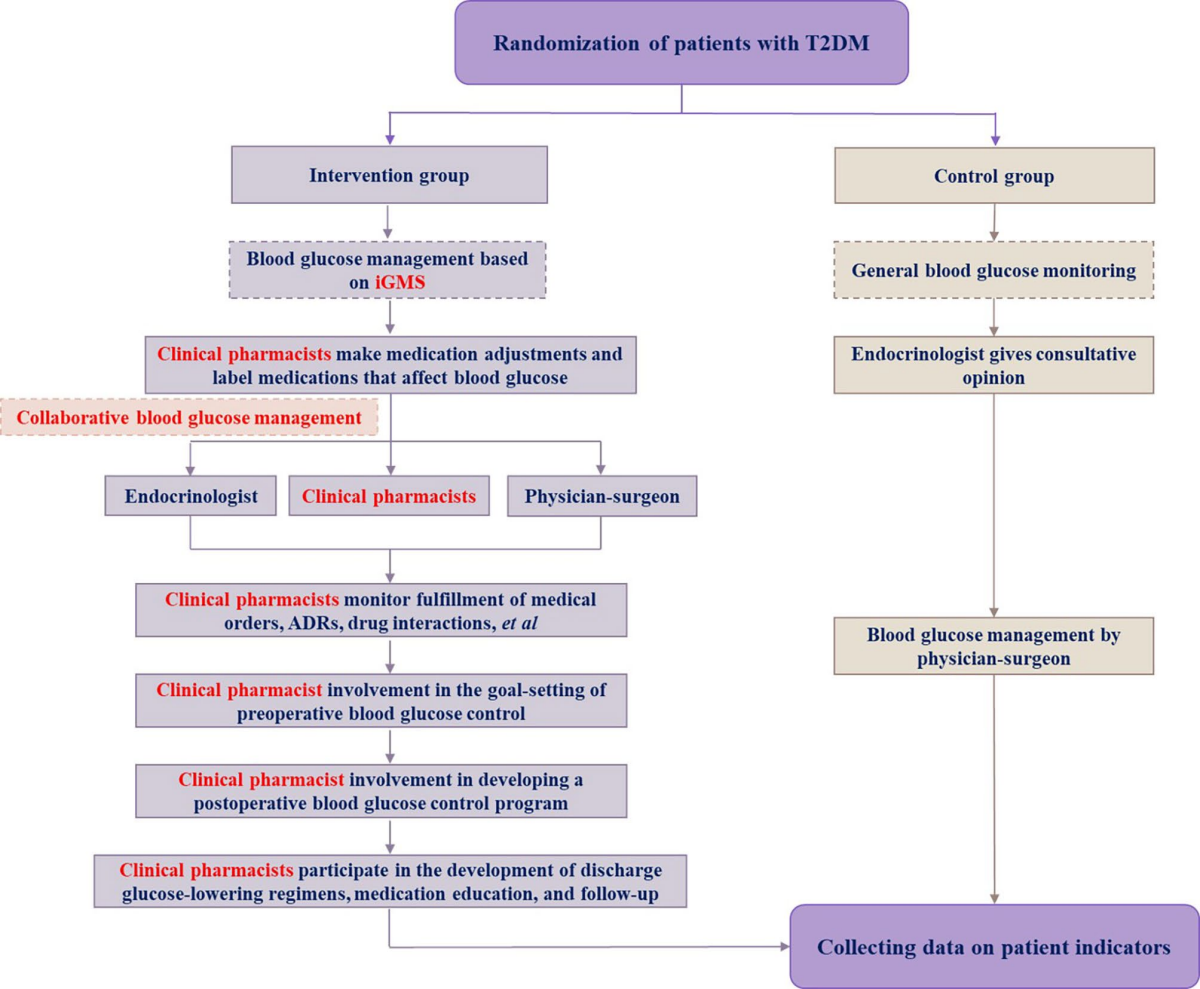
Specific management flows of two groups patients with T2DM. Abbreviations used: iGMS, informatized glucose management system; ADRs, adverse drug reactions

Clinical pharmacists were engaged in the whole process of the blood glucose management for perioperative patients under the guidance of expert consensus. Detailed duties include: (1) information collection before management, and **establishing** a blood glucose management form for patients, registering information about the treatment of patients and daily blood glucose monitoring level; (2) bedside checkups daily, medication treatment management, and **providing** guidance to patients about medication, diet, and exercise education; (3) **participating** in the determination of the date for surgery and type of surgery for patients, **specifying** the blood glucose control target for patients before surgery, and also **evaluating** the current level of blood glucose control to provide doctors with suggestions for medication; (4) monitoring of adverse drug reactions during treatment; (5) formulation **of** the management program and criteria; (6) creating strategies to lower blood glucose for patients who will be discharged from the hospital and providing education on medications. The specific management process is shown in Fig. 1.

### Responsibilities of clinical pharmacists

Clinical pharmacists are responsible for monitoring the blood glucose data of perioperative patients based on the iGMS. They are actively involved in the entire process of perioperative blood glucose management, guided by evidence-based medicine such as expert consensus and clinical guidelines. Clinical pharmacists participate in blood glucose management through the following main measures: (1) Clinical pharmacists begin by collecting information, conducting pharmaceutical interviews, and performing pharmaceutical assessments. They establish blood glucose management profiles for patients, record medication histories, and annotate drugs that can affect blood glucose. (2) Clinical pharmacists provide pharmaceutical care for patients with abnormal blood glucose levels. This includes tracking blood glucose monitoring results, reviewing prescriptions, and monitoring for adverse reactions using iGMS. If there is poor blood glucose control or changes in the patient’s condition during treatment, clinical pharmacists provide feedback to the physicians to make timely adjustments. (3) Clinical pharmacists, considering the patient’s individual condition, develop personalized management plans and guidelines for patients with abnormal blood glucose levels. (4) Clinical pharmacists participate in discussions about the patient’s surgical date and type of surgery. They also contribute to establishing preoperative blood glucose control goals. Additionally, they assess the patient’s current blood glucose control level and provide recommendations to physicians for adjusting medication treatment plans. (5) Clinical pharmacists offer medication guidance during the patient’s hospital stay and medication education upon discharge. This includes instructions on medication administration, prevention, and management of low blood sugar, among other topics.

### Collection of clinical index

Detailed history taking and physical examination **were** performed for each patient, and information on age, gender, height (in meters), weight (in kilograms), waist and hip circumferences (in centimeters), duration of T2DM, history of smoking and alcohol consumption, diabetic retinopathy (DR), dosage of insulin, systolic blood pressure (SBP), diastolic blood pressure (DBP), fasting plasma glucose (FPG), and postprandial plasma glucose (PPG) were recorded. Serum lipids including total cholesterol (TC), triglyceride (TG), low-density lipoprotein (LDL-c), and high-density lipoprotein (HDL-c) were detected using a Roche Cobas8000 analyzer (Roche, Basel, Switzerland) with standard laboratory methods. The levels of C-peptide were measured by an electrochemiluminescence assay (Roche, Shanghai, China). The amounts of glycated hemogolobin (HbA1c) were determined using the Variant II Turbo system (Bio-Rad, America). The general anthropometric parameters such as height, weight, waist circumference and hip circumference were measured in the morning on an empty stomach. Waist circumference was measured at the midpoint of the line connecting the lower rib cage and the skeleton, and hip circumference was measured at the level of the greater trochanter of the femur. Body index (BMI) and waist to hip ratio (WHR) were calculated. BMI = body weight (kg)/height (m)^2^, WHR = waist circumference (cm)/hip circumference (cm). Islet function was stratified into 3 grades of mild, moderate, and severe abnormalities according to the ratio of peak C-peptide to fasting C-peptide [22].

### Grading criteria for surgery

(1) Level I: various surgeries with low technical difficulty, simple surgical procedures, and low risk. (2) Level II: winter surgeries with average technical difficulty, uncomplicated surgical procedures, and moderate risk, (3) Level III: various surgeries with a relatively high level of technical difficulty, complicated surgical procedures, and moderate risk. (4) Level IV: various surgeries with high technical difficulty, complex surgical procedures, and high degree of risk.

### Evaluation indicators of effectiveness

(1) Preoperative glycemic compliance of patients: according to the blood glucose management control goals for perioperative patients recommended by the Chinese Expert Consensus on Glycemic Management in Hospitalized Patients [23], the preoperative blood glucose compliance rate was the percentage of the number of patients meeting the standard to the total number of patients; (2) preoperative waiting time was the time from the time patients received blood glucose management to the date of the operation; and (3) the number of days of inpatient.

### Indicators of adverse events

(1) Incidence of hypoglycemia: blood glucose ≤ 3.9 mmol/L is the judgment standard for hypoglycemic events, and the percentage of the number of patients with one or more hypoglycemic events to the total number of patients; (2) Incidence of hyperglycemia: the percentage of the number of patients with FPG > 7.8 mmol/L, and PPG or random blood glucose > 10.0 mmol/L to the total number of patients. (3) Determination of postoperative infection: the patients showed elevated body temperature, abnormal elevation of blood routine and C-reactive protein test at least 2 times after surgery. In addition, a comprehensive evaluation was made by combining the description of the local condition of the incision in the medical record with the diagnosis of infection at the operation area and the healing status of the incision. The percentage of the number of individuals meeting the above phenomena to the total number of patients is the incidence of postoperative infection.

### Statistical analysis

All data were expressed as mean ± standard deviation (Mean ± SD) or percentage as appropriate. Statistical analyses were performed using SPSS software (version 13.0 for Windows; SPSS Inc., Chicago, IL, USA). The paired t-test was used for intra-group comparisons of before and after management. The two-sample t-test was used to compare the characteristics between control group and intervention group. The Chi-square test was used for comparisons of counting data. Two-sided tests were used for all analyses, and *P* < 0.05 indicated statistically significant.

## Results

### Clinical and demographic characteristics of patients with T2DM

The general data of subjects in control group and intervention group included age, gender, history of smoking and alcohol consumption, combined DR, duration of T2DM, BMI, WHR, FPG, PPG, HbA1c, islet function grading, TC, TG, LDL-c, HDL-c, SBP, DBP, surgery level, and dosage of insulin. At baseline, there was no significant difference between control group and intervention group (*P* all > 0.05, Table 1). It indicates the comparability between the control group and intervention group.

**Table 1:** Baseline clinical characteristics of patients with type 2 diabetes mellitus in the control and intervention groups

Parameters	Control group	Intervention group	*P* value
N(men/women)	101/49	94/56	0.397
Age (years)	47.85 ± 12.26	44.38 ± 14.03	0.181
Smoking (yes/no)	32/118	30/120	0.776
Drinking (yes/no)	15/135	17/133	0.708
DR (yes/no)	47/103	52/98	0.539
Duration of T2DM	11.02 ± 5.07	11.03 ± 4.94	0.982
BMI (kg/m^2^)	28.19 ± 3.92	27.79 ± 3.76	0.374
WHR	0.94 ± 0.05	0.94 ± 0.05	0.504
FPG (mmol/L)	10.22 ± 3.50	10.81 ± 3.15	0.129
PPG (mmol/L)	15.67 ± 4.46	15.98 ± 4.30	0.538
HbA1c (%)	9.38 ± 1.79	9.55 ± 1.69	0.413
Islet function grading (n/%)			
Mild impairment	2 (2.94)	1 (1.39)	
Moderate impairment	21 (30.88)	20 (27.78)	
Severe impairment	45 (66.18)	51 (70.83)	0.734
TG (mmol/L)	2.37 ± 1.73	2.46 ± 1.78	0.682
TC (mmol/L)	4.80 ± 1.18	5.14 ± 1.42	0.028
HDL-c (mmol/L)	2.46 ± 1.78	2.46 ± 1.73	0.902
LDL-c (mmol/L)	3.05 ± 1.04	3.10 ± 1.22	0.734
SBP (mmHg)	133.23 ± 16.89	133.89 ± 18.42	0.747
DBP (mmHg)	84.23 ± 9.49	83.87 ± 10.71	0.762
Surgery level (n/%)			
Level I	12 (8.00)	15 (10.00)	
Level II	23 (15.33)	16 (10.67)	
Level III	32 (21.33)	30 (20.00)	
Level IV	83 (55.33)	89 (59.33)	0.601
Total daily insulin dose/weight (U/kg)	27.99 ± 10.26	29.07 ± 10.06	0.358

Abbreviations used: DR, diabetic retinopathy; T2DM, type 2 diabetes mellitus; BMI, body mass index; WHR, waist to hip ratio; FPG, fasting plasma glucose; PPG, postprandial plasma glucose; HbA1c, hemoglobin A1c; TG, triglyceride; TC, total cholesterol; HDL-c, high-density lipoprotein-cholesterol; LDL-c = low-density lipoprotein-cholesterol; SBP, systolic blood pressure; DBP, diastolic blood pressure. Note: Data are presented as mean ± standard deviation or n (%)

### Improvement of fasting plasma glucose and post plasma glucose

At baseline, there was no significant difference in FPG and PPG between the control group and intervention group (*P* > 0.05). On the third day after consultation, the control group showed a significant decrease in FPG and PPG (*P* < 0.05). On the third day after receiving blood glucose management, the FPG and PPG levels of T2DM patients in the clinical pharmacist management group were significantly reduced compared to the control group (*P* < 0.05), as shown in Table 2.

**Table 2:** Improvements of fasting blood glucose and postprandial blood glucose of patients with type 2 diabetes mellitus in the control and clinical pharmacist management groups

Parameters	Control group	Intervention group	*P* value
FPG-Day1 (mmol/L)	10.22 ± 3.50	10.81 ± 3.15	0.129
PPG-Day1 (mmol/L)	15.67 ± 4.46	15.98 ± 4.30	0.538
FPG-Day3 (mmol/L)	9.20 ± 3.15	7.57 ± 2.20	0.000
PPG-Day3 (mmol/L)	13.32 ± 3.79	10.86 ± 2.93	0.000

Abbreviations used: FPG, fasting plasma glucose; PPG, postprandial plasma glucose. Note: Data are presented as mean ± standard deviation

### Glucose fluctuations of patients with T2DM during the perioperative period

**Fig. 2:**
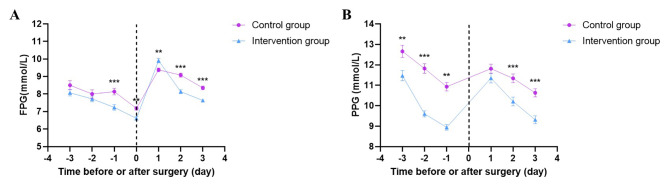
Comparison of blood glucose levels at Day1/2/3 before and after surgery between the control group (n = 150) and intervention group (n = 150). **A**: fasting plasma glucose (FPG), **B**: post plasma glucose (PPG). Control group compared with intervention group, ^* *^P < 0.01, ^***^P < 0.001

The FPG and PPG levels of patients were analyzed 3 days before surgery, on the day of surgery and 3 days after surgery respectively, so that the fluctuation of blood glucose of patients could be dynamically evaluated. Compared with the control group, the FPG of patients in the intervention group gradually decreased and fluctuated less, as well as the FPG of patients in the intervention group on the day of surgery was significantly lower (*P* < 0.05). Compared with the control group, the PPG of patients in the intervention group decreased more remarkable, and the difference was significant (*P* < 0.05) (Fig. 2, Table S1). It showed that the FPG and PPG levels were closer to the target in the intervention group than the control group.

### Comparison of indicators associated with management effectiveness

Compared with the control group, the preoperative glucose compliance rate was higher in the intervention group, and the difference was significant (60.67% vs. 35.33%, *P*<0.05), while the preoperative waiting time [(5.27 ± 3.34) vs. (7.45 ± 4.38), *P*<0.05] and length of hospitalization [(11.11 ± 4.56) vs. (14.87 ± 5.39), *P*<0.05] were significantly shorter in the intervention group, as shown in Table 3.

**Table 3:** Comparison of indicators related to the management effect of patients with type 2 diabetes mellitus in the control and clinical pharmacist management groups

Parameters	Control group	Intervention group	*P* value
Preoperative blood glucose compliance (n/%)	53 (35.33)	91 (60.67)	0.000
Preoperative waiting time (d)	7.45 ± 4.38	5.27 ± 3.34	0.000
Length of hospitalization (d)	14.87 ± 5.39	11.11 ± 4.56	0.000
HbA1c detection rate (n/%)	131 (87.33)	140 (93.33)	0.079
Blood glucose monitoring times daily	3.91 ± 2.20	4.98 ± 1.94	0.000

Abbreviations used: HbA1c, hemoglobin A1c. Note: Data are presented as mean ± standard deviation or n (%)

### Adverse events

Compared with the control group, after the blood glucose management was carried out by the clinical pharmacist via the iGMS, the incidence of hyperglycemia (32.00% vs. 62.67%, *P*<0.05) and hypoglycemia (8.67% vs. 18.00%, *P*<0.05) was lower, and the difference was significant. The incidence of postoperative infections of patients in the intervention group was less than that in the control group, but the difference was not significant (18.00% vs. 24.67%, *P* > 0.05), as shown in Table 4.

**Table 4:** Analysis of adverse events of patients with type 2 diabetes mellitus in the control and clinical pharmacist management groups

Parameters	Control group	Intervention group	*P* value
Incidence of hypoglycemia(n/%)	27 (18.00)	13 (8.67)	0.017
Incidence of hyperglycemia(n/%)	94 (62.67)	48 (32.00)	0.000
Postoperative infection rate(n/%)	37 (24.67)	27 (18.00)	0.159

Note: Data are presented as n (%)

## Discussion

Abnormal blood glucose levels of perioperative patients is an essential cause of clinical problems such as increased risk of infection, leading to non-healing of wounds and cardiovascular events, which prolong the duration of postoperative hospitalization and even allow to influence the long-term prognosis [24]. Therefore, scientific monitoring and management of blood glucose is one of the priorities of perioperative management. It has been shown that when standardized perioperative glucose management is given in a prompt manner, postoperative infections, reoperation rate, and the mortality rate will be significantly decreased [9, 25]. However, the current management situation of perioperative patients with T2DM **presents** the following problems: (1) insufficient frequency of blood glucose monitoring; (2) untimely blood glucose management in perioperative patients with T2DM; and (3) insufficient standardization of blood glucose regimen in perioperative patients with T2DM. **The** iGMS can realize automatic uploading, recording, archiving and analysis of data through the establishment of an in-hospital blood glucose management networking system. The unified management of blood glucose data in the whole hospital can detect problems in time, which can significantly improve the management effect and enhance the efficiency. On the other hand, it has been shown that the participation of clinical pharmacists, under the authorization of healthcare providers, can have a positive influence on clinical outcomes related to glycemic management [26, 27]. Accordingly, in order to promptly understand the blood glucose variation of patients in the perioperative period and adapt the blood glucose management regimen, a new model for clinical pharmacists to participate in perioperative blood glucose management via the iGMS was established in this study, and the effectiveness of the model was analyzed in the example of patients with T2DM, and the results showed that the model greatly enhanced the service value of the clinical pharmacists, and significantly optimized the effectiveness of blood glucose.

The iGMS can help clinical pharmacists effectively manage the blood glucose of surgical patients with T2DM in real time. Clinical pharmacists monitor the blood glucose of perioperative type 2 diabetes mellitus patients uploaded into the iGMS in real time, and in further combination with symptoms of the patient’s condition, individualized management advice is given. The inclusion of all patients who are hospitalized into the blood glucose management system is critical to improving the quality of medical care [28]. Jacobi et al. [29] demonstrated that clinical pharmacists are pharmacy professionals who can provide professional pharmacy services and comprehensive medication management for patients, and are important members of clinical care for patients. In addition, it is also widely acknowledged that clinical pharmacists provide patients with specialized medication guidance, which is instrumental in the improvement of blood glucose management compliance [30–32]. In our study, we constructed a new model of perioperative glycemic management involving clinical pharmacists based on the results of the above study, and we enhanced its efficiency and effectiveness by relying on the iGMS.

All subjects had lower blood glucose after 3 days of management compared to the time of admission, and blood glucose was significantly lower in the intervention group compared to the control group. Potential reasons for this are that benefiting from the iGMS, clinical pharmacists may be able to concern about abnormal blood glucose levels in a more immediate manner and provide patients with more specialized treatment advice and standardized diabetes health education. The dynamic blood glucose analysis before and after surgery revealed that the preoperative blood glucose compliance rate was 35.33% and 60.67% in the control and intervention groups, respectively, reaching a higher preoperative blood glucose compliance rate in the intervention group. It may be attributed to the continuous and dynamic management of patients in the intervention group. Clinical pharmacists can grasp the blood glucose levels of patients in a real-time manner via the iGMS, and provide timely feedback to doctors for individualized adjustment of glucose-lowering regimens and drug dosages. Confronted with elevated blood glucose due to postoperative stress, the clinical pharmacists took timely and proactive steps to assist the surgeons to formulate postoperative glucose-lowering programs according to the surgical anesthesia mode, type of surgery and control goals of the patients, thus the patients in the intervention group had smaller fluctuation of blood glucose in the perioperative period.

Patients in the intervention group had better control of blood glucose levels during the perioperative period and reduced preoperative waiting time and hospitalization time significantly, which is consistent with the results of previous studies [33]. Analyses for the occurrence of adverse events found lower rates of hyperglycemia and hypoglycemic events in the intervention group. Due to professional constraints, surgical physicians tend to worry excessively about patient hypoglycemia or ignore the adverse effects of hyperglycemia. The involvement of clinical pharmacists in perioperative blood glucose management not only increases the effectiveness of glycemic control, but also improves the indicators related to medical management.

This study also has some limitations, firstly, the sample size included is relatively small, which may result in some meaningful clinical phenomena not being observed. Second, this study examined the effect of blood glucose management and some outcome indicators in perioperative patients, but failed to observe other metabolisms such as blood pressure and lipids; And third, it only focused on blood glucose changes in the perioperative period, but not on long-term management indicators. Therefore, follow-up of patients after discharge will be conducted in subsequent studies to more comprehensively assess the benefits to patients, with a view to constructing a better model of perioperative blood glucose management.

## Conclusion

In view of the above, the perioperative blood glucose management system established in this study based on the iGMS with the participation of clinical pharmacists has obvious advantages for regulating blood glucose in perioperative patients, which can more effectively improve FPG, PPG, and attainment of the target, and shorten the preoperative waiting time and the length of hospitalization, which is of great significance for improving the management of the patients in the perioperative period.

### Electronic supplementary material

Below is the link to the electronic supplementary material.

Supplementary Material 1

## Data Availability

All data generated during this study are included in this published article and its supplementary information files.

## List of abbreviations

iGMS: Informatized glucose management system
T2DM: Type 2 diabetes mellitus
WHO: World health organization
ADRs: Adverse drug reactions
DR: Diabetic retinopathy
FPG: Fasting plasma glucose
PPG: Postprandial plasma glucose
HbA1c: Hemoglobin A1c
SBP: Systolic blood pressure
DBP: Diastolic blood pressure
TC: Total cholesterol
TG: Triglyceride
LDL-c: Low-density lipoprotein
HDL-c: High-density lipoprotein
BMI: Body index
WHR: Waist to hip ratio
