# Supplementary material for: Evaluation of the effectiveness regarding the participation of pharmacists in perioperative blood glucose management via the iGMS: a pilot RCT
Source: Diabetol Metab Syndr. 2023 Nov 17;15:236. doi: 10.1186/s13098-023-01221-8 (PMC10656936; doi:10.1186/s13098-023-01221-8)
Supplement: Supplementary file 1 — Supplementary Material 1 [file 13098_2023_1221_MOESM1_ESM.docx]

**Table S1** Blood glucose levels at Day 1/2/3 before and after surgery of patients with type 2 diabetes mellitus in the control and clinical pharmacist management groups

| **Parameters** | **Control group** | **Intervention group** | ***P* value** |
| --- | --- | --- | --- |
| FPG-surgery D-3 (mmol/L) | 8.5±3.15 | 8.07±2.20 | 0.168 |
| PPG--surgery D-3 (mmol/L) | 12.66±3.60 | 11.47±3.07 | 0.002 |
| FPG-surgery D-2 (mmol/L) | 8.00±2.95 | 7.72±1.99 | 0.339 |
| PPG--surgery D-2 (mmol/L) | 11.82±2.87 | 9.60±1.85 | 0.000 |
| FPG-surgery D-1 (mmol/L) | 8.14±2.29 | 7.24±1.97 | 0.000 |
| PPG--surgery D-1 (mmol/L) | 10.93±2.52 | 8.94±1.71 | 0.000 |
| FPG-surgery D0 (mmol/L) | 7.18±1.29 | 6.61±1.41 | 0.001 |
| FPG-surgery D1 (mmol/L) | 9.38±1.35 | 9.91±1.61 | 0.002 |
| PPG--surgery D1 (mmol/L) | 11.81±2.72 | 11.35±2.92 | 0.164 |
| FPG-surgery D2 (mmol/L) | 9.08±1.39 | 8.13±1.32 | 0.000 |
| PPG--surgery D2 (mmol/L) | 11.34±2.61 | 10.21±2.63 | 0.000 |
| FPG-surgery D3 (mmol/L) | 8.35±1.24 | 7.64±1.27 | 0.000 |
| PPG--surgery D3 (mmol/L) | 10.63±2.45 | 9.31±2.40 | 0.000 |

Abbreviations used: FPG, fasting plasma glucose; PPG, postprandial plasma glucose.

Note: Data are presented as mean±standard deviation.
